# Study of the Optical and Acoustic Parameters and Surface Tensions of 3,4,4′-Trichlorodiphenylurea in Binary Mixtures with Different Organic Solvents between (293.15 and 323.15) K

**DOI:** 10.3390/molecules29194521

**Published:** 2024-09-24

**Authors:** Florinela Sirbu, Alina Catrinel Ion, Ion Ion

**Affiliations:** 1 “Ilie Murgulescu” Institute of Physical Chemistry of Romanian Academy, Department of Chemical Thermodynamics, 202 Splaiul Independentei Str., 060021 Bucharest, Romania; 2 Department of Analytical Chemistry and Environmental Engineering, Faculty of Chemical Engineering and Biotechnologies, National University of Science and Technology POLITEHNICA Bucharest, 1-7 Polizu Str., 011061 Bucharest, Romania; ion.ion@upb.ro

**Keywords:** physicochemical properties, 3,4,4′-Trichlorodiphenylurea, organic solvents, solvation number, surface tension, liquid mixtures

## Abstract

In the present investigations, the density, refractive index and speed of sound for pure organic solvents and binary liquid mixtures of 3,4,4′-Trichlorodiphenylurea between (293.15 and 323.15) K temperatures have been measured up to the solubility limit. From these experimental results, the acoustic impedance, the isentropic compressibility coefficient, the space-filling factor, the specific refraction, the relaxation strength, the intermolecular free length, the surface tension, the solubility and the solvation number of triclocarban in six organic solvents, namely ethyl alcohol, n-Propyl alcohol, n-Butyl alcohol, Tetrahydrofuran, N,N-Dimethylformamide and N,N-Dimethylacetamide have been computed. The studied acoustic and optical parameters and surface tension behavior versus temperature in pure solvents and binary mixtures were useful in understanding the nature and the extent of interaction between the solute and solvent molecules. The results also show the presence of higher degree of interaction between triclocarban and nitrogen-containing solvents in comparison with other solvents. The distribution of triclocarban in water/organic solvent mixtures is frequently encountered in wastewater treatment plants.

## 1. Introduction

Study of the thermophysical parameters of environmental contaminants in different organic solvents contributes to understanding of physicochemical behavior in liquid mixtures. The study of miscibility and the molecular interactions in liquid solvent mixtures are useful in industrial, biological and environmental processes, with a high practical importance [1,2,3].

The 3,4,4′-Trichlorodiphenylurea compound, known by the name of triclocarban (TCC), is a synthetic antibacterial agent used in very low concentrations as an antiseptic in cosmetic and health consumer products [4,5,6]. Having a low solubility in water, triclocarban can be introduced into all manufactured products only dissolved in organic solvents [7,8]. Triclocarban has begun to be considered one of the contaminants whose presence must be detected and quantified above certain limits in aqueous polluted media in the last years [1,9]. A special interest is worth noting in the study of the thermodynamic behavior of mixtures containing TCC and organic solvents, which are important in the processes of TCC detection and elimination from polluted water environments [1,9,10,11,12]. The available experimental studies regarding TCC dissolution in organic solvents [3,10,11,12,13] are very few, even if the interactions of this environmental contaminant with organic solvents [14,15] give information about solubility/miscibility, important in the risk characterization of emerging pollutants in environmental matrices [16,17]. The applied technologically and economically effective and feasible methods for the removal of contaminants from industrial wastewater systems include precipitation, solvent extraction, biodegradation, chemical oxidation, evaporation, membrane filtration, ion-exchange, carbon adsorption and electrochemical approaches [17,18,19,20,21,22,23,24,25].

Although the number of studies in the literature on TCC in solvents mixtures is increasing [4,18], there is still a lack of experimental data on their thermophysical and acoustical behavior and on the description of molecular interactions between solute and solvent [4]. Triclocarban is a hydrophobic compound whose molecule presents stronger interactions with certain functional groups from organic solvents, causing higher degrees of interaction and better solubilities. Its solubility was studied in different organic solvents, like alcohols (ROHs), chloroform and heptane, at different temperatures [26,27].

The chemical solubility and partitioning behavior are key input parameters in most organic contaminant environmental fate and transport models used to screen chemicals for human and environmental health risks. Measured values of TCC solubility and K_ow_ from the literature present important differences, based on the methods of determination [18,28,29]. 

In this work, as a continuation of our previous liquid mixtures studies [30,31,32,33,34], we evaluate the density, refractive index and speed of sound of TCC contaminant in six organic solvents, namely Ethyl alcohol (EA), n-Propyl alcohol (nP), n-Butyl alcohol (nB), Tetrahydrofuran (THF), N,N-Dimethylformamide (DMF) and N,N-Dimethylacetamide (DMA), for which experimental data are not available. Experimental data were measured close to the solubility limit in pure solvents and in binary mixtures at seven temperatures between (293.15 and 323.15) K and at ambient pressure.

From measured experimental data, the acoustic impedance (Z), the isentropic compressibility coefficient (κ_S_), the space-filling factor (S), the specific refraction (r_D_), the relaxation strength (r), the intermolecular free length (L_f_), the surface tension (σ), the modified surface tension (σ_mod_) and the solvation number (Sn) of triclocarban in all the studied organic solvents have been computed and then correlated, as a function of temperature for pure solvents and binary solutions, by polynomial-type equations. 

The behavior of the acoustic and optical parameters and of the surface tension at the studied temperatures was useful in understanding the nature and the extent of interaction between the unlike molecules of solute and solvents. 

Thus, the present study on the thermophysical, optical and surface tension properties in binary mixtures of TCC and six organic solvents are very important for these applications in the manufacturing process of personal care products in the cosmetic industry [35].

## 2. Results

### 2.1. Tables and Figures

The measured values of densities, speeds of sound and refractive indices for pure Ethyl alcohol, n-Propyl alcohol, n-Butyl alcohol, Tetrahydrofuran, N,N-Dimethylformamide and N,N-Dimethylacetamide organic solvents are presented in Table 1 for all temperatures between (293.15 and 323.15) K compared with values available in the literature, at 298.15 K [36,37,38,39,40,41,42,43,44,45,46,47,48,49,50,51,52,53,54,55,56,57,58,59,60,61,62,63,64,65,66,67,68,69,70,71,72,73,74,75,76,77,78,79,80,81,82,83,84,85,86,87,88,89,90,91,92], together with the standard and combined uncertainties, which affected the experimental measurements for density (*ρ*), refractive index (n_D_) and speed of sound (u), respectively. 

The values of density, refractive index, speed of sound and solvation number are presented in Table 2, together with the X_1_ molar fraction of TCC and the specific estimated uncertainties.

In Table 3, the acoustic impedance, the isentropic compressibility coefficient, the space-filling factor, the specific refraction, the relaxation strength, the intermolecular free length and the surface tension for TCC-solvents binary mixtures are presented.

Table 4 shows the physicochemical parameters of TCC in organic solvents solubility (*s*) and (K_OW_) values at 298.15 K and at pressure p = 0.1 MPa

### 2.2. Formatting of Mathematical Components

The molal solvation number was calculated from binary mixtures and pure solvent isentropic compressibility data using the following equation [93]:

S_n_ = n_2_/n_1_ (1 − κ/κ_0_) (1)

where:

S_n_—solvation number of binary mixture;
n_2_—the number of moles of solvent in the sample;
n_1_—the number of moles of solute in the sample;
κ—isentropic compressibility of binary mixture;
κ_0_—isentropic compressibility of pure solvent.

**Figure 1:**
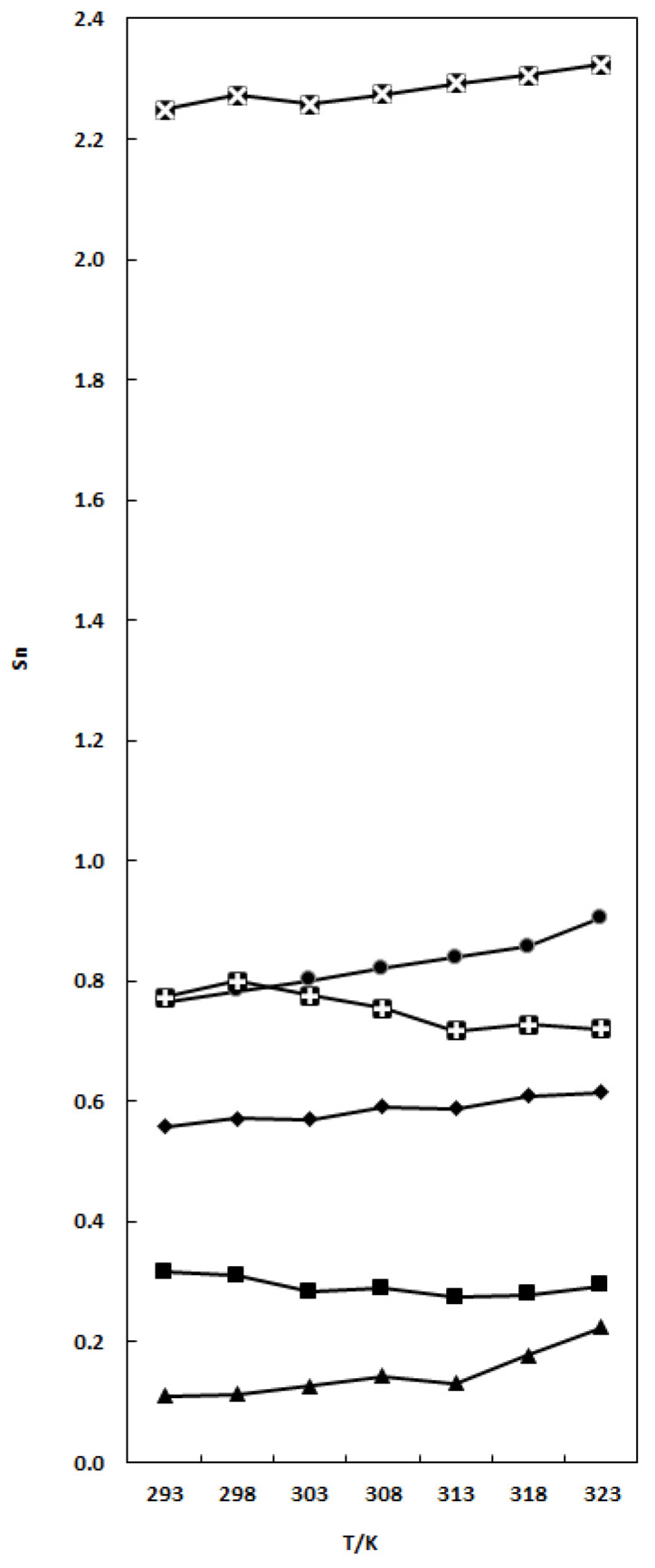
Solvation number versus temperature for TCC in binary TCC (1) + EA(2) (●), X_1_ = 0.0626; TCC (1) + nP (2) (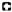), X_1_ = 0.0663; TCC (1) + nB (2) (■), X_1_ = 0.0697; TCC (1) + THF (2) (▲), X_1_ = 0.0390; TCC(1) + DMF (2) (♦), X_1_ = 0.1261; and TCC (1) + DMA (2) (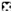), X_1_ = 0.0564 mixtures. The lines in the figure are presented only to visualize the Sn variation.

The molal solvation number is presented in Table 2 and Figure 1. 

The following thermodynamic acoustical and optical properties were estimated using the standard relations. The acoustic impedance (Z) was calculated using the following relation [94]:

Z = ρ u(2)

where:

ρ—the density (kg·m^−3^) in the mixture;
u—the speed of sound (m·s^−1^) in the mixture.

The κ_S_ isentropic compressibility coefficient for the pure solvent and binary mixtures has been calculated from the density data and the speed of sound using the Laplace relation [95].

κ_S_ = 1/ρ u^2^ (3)

The space-filling factor (S) was estimated from refractive index data (sodium D line) using the following relation, according to the method of Gerecze [96] and Lorentz–Lorenz [97,98]:

S = (n_D_^2^ − 1)/(n_D_^2^ + 2) (4)

where n_D_ is the refractive index of the binary solution. 

The specific refraction (*r_D_*) was computed from the density and space-filling factor (*S*) values using the Lorentz and Lorenz equation [87]:

r_D_ = (1/ρ) (n_D_^2^ − 1)/(n_D_^2^ + 2) (5)

The relaxation strength (*r*) was estimated using the following relation [98]:

r = 1 − u^2^/u_ct_^2^ (6)

where:

u—the speed of sound in the experimental solution;
u_ct_—a constant with a value of 1600 m∙s^−1^ [99].

The intermolecular free length of liquids was calculated from compressibility values of binary mixtures, with and without TCC, with following empirical relation [100,101,102]:

L_f_ = K′ κ_S_^0.5^ (7)

where:

K′—a temperature dependent constant, with the name Jacobson’s constant [101]:
K′ = (93.875 + 0.375 T) 10^−8^
T—absolute temperature;
κ_S_—the isentropic compressibility in binary mixtures.

Surface tension and modified surface tension for the studied binary mixtures, with and without TCC, have been calculated from density and speed of sound data with the equations [35,103]:

σ = 6.3 10^−4^ ρ u^3/2^ (8)

σ_mod_ = 10^−4^ T^1/3^ ρ u^3/2^ (9)

where:

ρ—density;
u—speed of sound;
T—absolute temperature;

From the TCC solubility of the prepared samples of TCC with six organic solvents, using back-calculation, K_OW_ (the octanol/water partition constant, reflecting the lipophilicity or hydrophobicity of an organic compound), is estimated from the below equation [16]:

log (s) = 0.693 − 0.96 log (K_ow_) − 0.0092 (t_m_ − 25) − 0.00314 M_TCC_ (10)

where:

s—solubility from prepared samples, measured in [mol L^−1^];
t_m_—the TCC melting point; t_m_ = 250 °C;
M_TCC_—the TCC molecular mass; M_TCC_ = 313.58 [g mol^−1^].

The values for *s* solubility and K_OW_ constant of the physicochemical parameters are presented in the Table 4.

## 3. Discussion

The obtained experimental data of thermophysical properties (Table 1) for Ethyl alcohol, n-Propyl alcohol, n-Butyl alcohol, Tetrahydrofuran, N,N-Dimethylformamide and N,N-Dimethylacetamide pure solvents used in binary mixtures with TCC solute were in agreement with the literature [36,37,38,39,40,41,42,43,44,45,46,47,48,49,50,51,52,53,54,55,56,57,58,59,60,61,62,63,64,65,66,67,68,69,70,71,72,73,74,75,76,77,78,79,80,81,82,83,84,85,86,87,88,89,90,91,92].

The values of density, refractive index and speed of sound (Table 2) are observed to decrease by increasing temperature and by adding TCC solute to the mixture. The density variation is in following order: EA < nP < nB < THF < DMA < DMF, similarly with the variation in the speed of sound. The density for binary mixtures is higher than for pure solvents. 

The refractive index values increase for pure DMF and DMA solvents versus refractive index values in binary mixtures. The structural influences of organic solvents over the molecules of environmental contaminants may be explained in this context based on the different variations in refractive indices.

From Table 2 and Figure 1, the solvation number values are found to decrease for nP and nB and increase by increasing temperature for the other four solvents. 

The Sn variation of TCC in organic solvents is the following: THF < nB < DMF < nP < EA < DMA. The lower S_n_ values obtained may be due to a reduced interaction of TCC solute with the solvent. 

The change in the isentropic compressibility coefficient is only due to the variation in the structural arrangement of the molecules because this depends only on the speed of sound and on the density of the solution and not on the temperature [35].

The values of the adiabatic compressibility are positive, indicating that the bulk solvent molecules are poorly compressible in comparison with the solvent molecules present around the primary and secondary solvation shell of TCC, inducing weak interactions [104,105,106].

Table 3 shows that by increasing the addition of TCC and by increasing temperature, the κ_S_ isentropic compressibility coefficient, r relaxation strength and L_f_ intermolecular free length are observed to increase, but the Z acoustic impedance and the S space-filling factor decrease in the mixture.

The change observed in Z by adding TCC is attributed to a change in the speed of sound in the mixture. This behavior is due to the association of the molecules and the formation of molecular aggregates.

The change in Z and S with temperature and the adding of the TCC compound may be interpreted in terms of an increase in intermolecular forces and a subsequent decrease in the relaxation of the molecules. 

The intermolecular free length (L_f_) of liquids is the distance between the surfaces of neighboring molecules, a very important parameter for the investigation of the nature and strength of the interactions [107].

**Figure 2:**
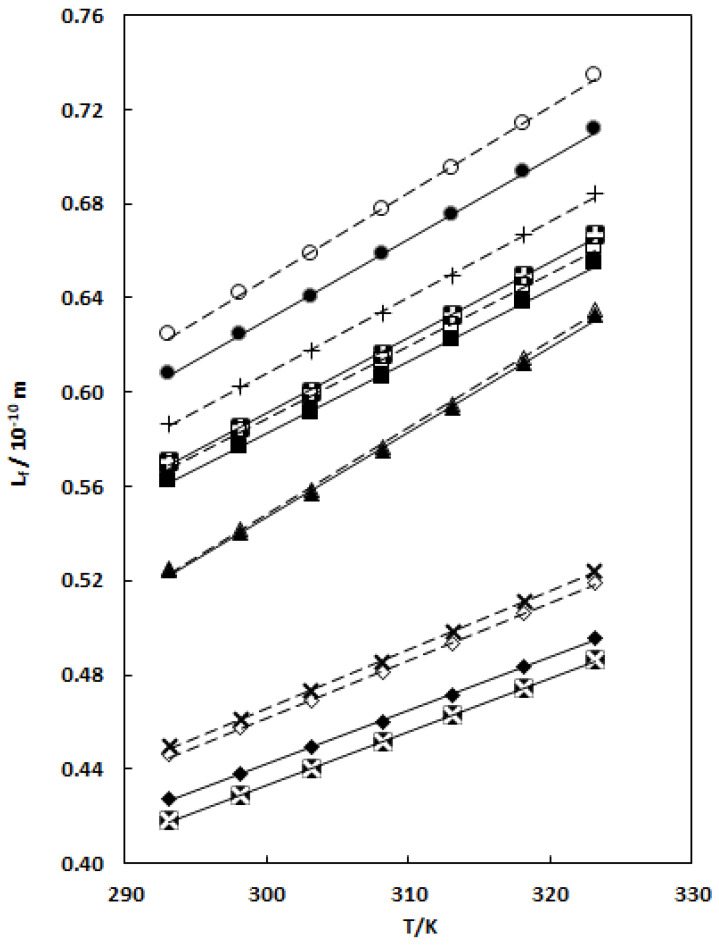
Intermolecular free length versus vs. temperature for pure EA (○), X_1_ = 0; nP (+), X_1_ = 0; nB (□), X_1_ = 0; THF (Δ), X_1_ = 0; DMF (◊), X_1_ = 0; and DMA (×), X_1_ = 0 solvents and for TCC in binary TCC (1) + EA (2) (●), X_1_ = 0.0626; TCC (1) + nP (2) (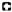), X_1_ = 0.0663; TCC (1) + nB (2) (■), X_1_ = 0.0697; TCC (1) + THF (2) (▲), X_1_ = 0.0390; TCC (1) + DMF (2) (♦), X_1_ = 0.1261; and TCC (1) + DMA (2) (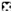), X_1_ = 0.0564 mixtures. – – linear correlation for pure solvents; ⎯⎯⎯ linear correlation for binary mixtures.

Table 3 and Figure 2 show that the intermolecular free length (*L*_*f*_) values increase with increasing temperature for all binary systems, in the following order:

DMA < DMF < THF < nB < nP < EA.

The behavior for κ_S_ and L_f_ is similar, in the same order.

The intermolecular free length (*L*_*f*_) values for binary mixtures are less than the *L*_*f*_ for pure solvents. The increase in L_f_ implies an increase in the number of free ions showing the occurrence of ionic dissociation [108,109]. 

**Figure 3:**
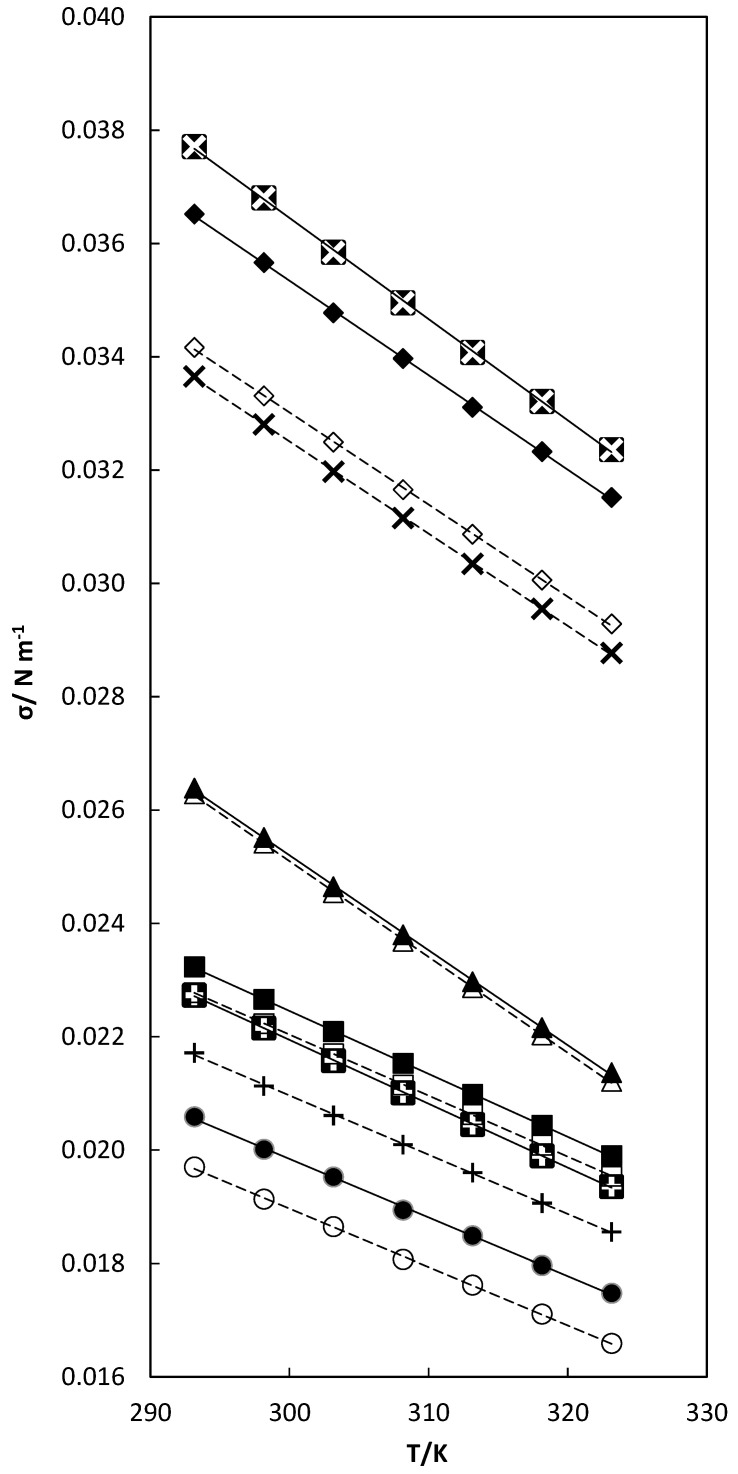
Surface tension versus temperature for pure EA (○), X_1_ = 0; nP (+), X_1_ = 0; nB (□), X_1_ = 0; THF (Δ), X_1_ = 0; DMF (◊), X_1_ = 0; and DMA (×), X_1_ = 0 solvents and for TCC in binary TCC (1) + EA(2) (●), X_1_ = 0.0626; TCC (1) + nP (2) (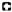), X_1_ = 0.0663; TCC (1) + nB (2) (■), X_1_ = 0.0697; TCC (1) + THF (2) (▲), X_1_ = 0.0390; TCC(1) + DMF (2) (♦), X_1_ = 0.1261; and TCC (1) + DMA (2) (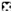), X_1_ = 0.0564 mixtures. – – linear correlation for pure solvents; ⎯⎯⎯ linear correlation for binary mixtures.

The values of surface tension and modified surface tension for the studied temperatures are presented in Table 3 and Figure 3.

The surface tension decreased linearly with increasing temperature [110,111]. As the temperature increases, the molecules of the solute and solvent are weakly bound and the kinetic energy of the molecules increases, resulting in a decrease in the cohesive forces between the molecules.

Surface tension increases for binary mixtures with the adding of TCC solute because of intermolecular separation, which appears by diluting the sample, resulting in stronger interactions based on intermolecular forces between functional groups from triclocarban and those of the studied solvents [12,112,113]. Surface tension in binary mixtures varies in the following order: Ethyl alcohol (EA) < n-Propyl alcohol (nP) < n-Butyl alcohol (nB) < Tetrahydrofuran (THF) < N,N-Dimethylformamide (DMF) < N,N-Dimethylacetamide (DMA). The modified surface tension varies in a similar way with the surface tension for all samples at all temperatures.

Table 4 presents the behavior of the (*s*) solubility and (K_OW_) constants of TCC in organic solvents at 298.15 K and at pressure p = 0.1 MPa. The two s and K_OW_ obtained physicochemical parameters show the importance of the solubility experimental data for characterizations of bioavailability and bioaccumulation [18,28,114,115,116] potential between TCC solute and studied organic solvents.

Chemical solubility and partitioning behavior are key input parameters in many transport models of environmental organic contaminants. These are used to screen chemicals for human and environmental health risks. Measured values of TCC solubility and Kow from the literature [18,28,114,115,116] present important differences, based on the methods of determination. It is very important to study the kinetics of the process before measuring in order to obtain the equilibrium concentration of the TCC in the studied solvents. 

## 4. Materials and Methods

### 4.1. Materials

The Triclocarban (TCC) Contaminant Was Provided by Sigma Aldrich (St. Louis, MI, USA). The organic Ethyl alcohol, n-Propyl alcohol and n-Butyl alcohol solvents have been supplied by Sigma Aldrich, the solvent N,N-Dimethylformamide from Merck, and Tetrahydrofuran and N,N-Dimethylacetamide were supplied by Fluka Chemie AG (Buchs, Switzerland), as presented in Table 5. All compounds were used without any pre-treatment because of their mass fraction purity higher than 0.95. The details of the chemicals used for sample preparation are presented in Table 1. The TCC/solvent experimental solutions were prepared by weight at a temperature of 298.15 K to a volume of approx. 25 g each, for reducing errors. The TCC solute needed for each sample was weighed with a Mettler-Toledo microbalance with a precision of ±2 × 10^−6^ g. 

### 4.2. Apparatus and Measurement Procedure

The experimental data for density and speed of sound of the pure solvents and binary mixtures were measured with an Anton Paar DSA 5000 digital (Wien, Austria) “Density and Sound Velocity Analyzer”, with a precision of ±10^−6^ g·cm^−3^**.** The temperature of the sample during the density measurements was controlled with a precision of ±10^−3^ K, with several Peltier units being used. The density and speed of sound values obtained for air and twice distilled, de-ionized and degassed water by calibration were reproducible within ±5 × 10^−6^ g·cm^−3^ and ±5 × 10^−2^ m·s^−1^, respectively. The specific conductance for water used for calibration was 5 × 10^−5^ S·m^−1^ at 298.15 K, according to the recommendations of the manufacturer.

Refractive indices of pure solvents and binary mixtures at the six temperatures were measured at sodium D-line, λ_D_ = 589.3 nm, using a digital automatic refractometer (Anton Paar RXA 170, Wien, Austria) with a precision of ±0.01 K in temperature and of ±0.000001 for refractive index. The refractometer was calibrated with the certified reference liquid (CRM) tetrachloroethylene and was further checked out by measuring the refractive index of doubly distilled, deionized water at atmospheric pressure. The obtained value for water refractive index was 1.33249 at 298.15 K, in good agreement with the literature [69,117].

## 5. Conclusions

The present study reports new experimental measurements of the ρ density, nD refractive index and u speed of sound of binary mixtures in six different organic solvents, with and without TCC, at seven different temperatures between (293.15 and 323.15) K, and at atmospheric pressure.

The obtained experimental results were used to calculate derived parameters such as acoustic impedance, isentropic compressibility coefficient, space-filling factor, specific refraction, relaxation strength, intermolecular free length, surface tension, solubility and solvation number

It is also observed that the derived estimated parameters for the binary mixtures containing TCC, an emerging environmental pollutant, behave similarly the pure organic solvents.

The trends in the variation in the optical and acoustic parameters and surface tensions indicate the existence of positive molecular interactions between the 3,4,4′-Trichlorodiphenylurea contaminant and the organic solvents from the studied binary mixtures.

The variations in the computed parameters also show a higher degree of interaction between 3,4,4′-Trichlorodiphenylurea and nitrogen-containing solvents such as N,N-Dimethylformamide and N,N-Dimethylacetamide compared to mixtures containing the others studied solvents.

## Figures and Tables

**Table 1:** Experimental values of densities (ρ), the speed of sound (u) and refractive index (n_D_) for pure Ethyl alcohol, n-Propyl alcohol, n-Butyl alcohol, Tetrahydrofuran, N,N-Dimethylformamide and N,N-Dimethylacetamide solvents at temperatures between (293.15 and 323.15) K and at pressure p = 0.1 MPa) along with available values at 298.15 K.

T, K	ρ, g cm^−3^	u, m s^−1^	*n*_D_
**Ethyl alcohol**			
293.15	0.789811	1161.63	1.361447
298.15	0.785489	1143.42	1.359439
	0.78506 [36]	1143.17 [36]	1.359130 [36]
	0.78511 [37]	1143 [37]	1.35922 [38]
	0.7855 [39]	1143.07 [40]	1.3593 [41]
	0.7893 [42]	1144 [43]	1.3605 [44]
	0.786 [45]	1142 [46]	1.360 [45]
303.15	0.781122	1128.18	1.357238
308.15	0.776707	1108.97	1.355301
313.15	0.772236	1094.72	1.353645
318.15	0.767711	1077.64	1.351281
323.15	0.761515	1061.53	1.349268
**n-Propyl alcohol**			
293.15	0.804317	1224.61	1.385059
298.15	0.800413	1206.42	1.383034
	0.800 [43,45,47]	1208.03 [45]	1.38307 [48]
	0.80021 [49]	1207.3 [47]	1.38370 [38]
	0.799657 [50]	1206 [48]	1.3832 [41]
	0.7996 [51]	1205.69 [52]	1.383 [45,47,53]
	0.79977 [54]	1209.4 [46]	1.3840 [55]
303.15	0.796251	1190.63	1.380950
308.15	0.792497	1174.36	1.378912
313.15	0.788632	1158.86	1.376804
318.15	0.784748	1141.31	1.374651
323.15	0.781084	1124.35	1.372472
**n-Butyl alcohol**			
293.15	0.809863	1258.46	1.398898
298.15	0.806857	1241.25	1.396938
	0.806 [47,56,57]	1241.8 [47]	1.397 [47]
	0.8071 [58]	1241 [59]	1.3967 [41]
	0.8070 [60]	1238.99 [61]	1.3972 [62]
	0.80590 [48,63]	1240.37 [64]	1.39702 [48]
	0.8053 [65]	1240.5 [63]	1.3983 [55]
303.15	0.802061	1226.28	1.394814
308.15	0.798782	1208.71	1.392757
313.15	0.795437	1192.14	1.390715
318.15	0.791082	1175.49	1.388654
323.15	0.786328	1158.65	1.386560
**Tetrahydrofuran**			
293.15	0.887551	1302.54	1.407321
298.15	0.882123	1278.86	1.404612
	0.882322 [66]	1278.93 [66]	1.4053 [66]
	0.88216 [67]	1279.38 [67]	1.40464 [67]
	0.88207 [68]	1277.60 [69]	1.4049 [70]
	0.882150 [71]	1280.1 [71]	1.405 [72]
	0.8828 [73]	1278 [72,73]	1.4037 [74]
303.15	0.876542	1254.62	1.40205
308.15	0.871126	1230.31	1.39938
313.15	0.865659	1206.87	1.39679
318.15	0.860174	1182.26	1.39415
323.15	0.854689	1157.73	1.39136
**N,N-Dimethylformamide**			
293.15	0.949789	1482.82	1.430508
298.15	0.945014	1462.94	1.428251
	0.944290 [75]	1458 [73]	1.4305 [76]
	0.94502 [77]	1457 [72]	1.429 [72]
	0.9445 [78,79]	1457.50 [80]	1.4290 [81]
	0.943978 [82]	1457.69 [75]	1.42810 [83]
	0. 95010 [84]	1463.76 [77]	1.42805 [77]
303.15	0.940119	1443.86	1.425988
308.15	0.935320	1423.79	1.423685
313.15	0.930455	1404.95	1.421411
318.15	0.925848	1384.87	1.419096
323.15	0.921060	1365.71	1.416780
**N,N-Dimethylacetamide**			
293.15	0.941052	1477.02	1.438107
298.15	0.936459	1456.92	1.435740
	0.9364 [85]	1453.68 [36]	1.43621 [86]
	0.9365 [80]	1455.37 [87]	1.43571 [88]
	0.93639 [86]	1475.3 [86]	1.4359 [89]
	0.9366 [90]	1458 [90]	1.435794 [36]
	0.93634 [91]	1478.98 [91]	1.4364 [92]
303.15	0.931848	1436.87	1.433288
308.15	0.927228	1416.83	1.430911
313.15	0.922604	1396.87	1.428593
318.15	0.917991	1377.03	1.426357
323.15	0.913344	1357.23	1.424067

Standard uncertainties u are as follows: u(T) = 0.02 K and u(p) = 0.04 MPa, and the combined expanded uncertainty Uc in mole fraction, density, speed of sound and refractive index was Uc(x) = 0.0008, Uc(ρ) = 0.004 g cm^−3^, Uc(u) = 1.2 m s^−1^ and Uc(n_D_) = 0.0005, respectively (0.95 level of confidence).

**Table 2:** Experimental values of densities (ρ), the ultrasonic velocities (u), refractive index (n_D_), together with the solvation number computed for binary TCC + solvent mixtures at temperatures between (293.15 and 323.15) K.

T, K	ρ/g cm^−3^	u/m s^−1^	*n*_D_	*Sn*	ρ/g cm^−3^	u/m s^−1^	*n*_D_	*Sn*
	**TCC (1) + AE (2), X_1_ = 0.0626**				**TCC (1) + nP (2), X_1_ = 0.0663**			
293.15	0.804476	1181.60	1.362256	0.76546	0.815279	1251.19	1.385218	0.77326
298.15	0.800156	1163.77	1.360200	0.78383	0.811173	1233.97	1.383165	0.80024
303.15	0.795834	1148.91	1.358040	0.80216	0.807021	1216.66	1.381185	0.77601
308.15	0.791388	1130.08	1.356017	0.82146	0.802821	1199.38	1.379087	0.75493
313.15	0.786860	1116.21	1.354388	0.83846	0.798572	1182.13	1.377012	0.71732
318.15	0.782251	1099.5	1.352035	0.85661	0.794268	1164.96	1.374886	0.72788
323.15	0.777548	1083.78	1.350055	0.90447	0.789907	1147.79	1.372540	0.72015
	**TCC (1) + nB (2), X_1_ = 0.0697**				**TCC (1) + THF (2), X_1_ = 0.0390**			
293.15	0.815711	1269.05	1.399069	0.31589	0.889290	1304.19	1.407681	0.11042
298.15	0.811823	1252.07	1.397035	0.30989	0.883858	1280.53	1.404894	0.11252
303.15	0.807897	1235.04	1.395032	0.28368	0.878381	1256.54	1.402220	0.12673
308.15	0.803933	1218.06	1.392923	0.28829	0.872861	1232.66	1.399653	0.14271
313.15	0.799931	1201.18	1.390879	0.27397	0.867305	1208.94	1.397029	0.13097
318.15	0.795882	1184.37	1.388827	0.27865	0.861711	1185.49	1.394445	0.17787
323.15	0.791782	1167.56	1.386741	0.29344	0.856069	1162.10	1.391881	0.22448
	**TCC (1) + DMF (2), X_1_ = 0.1261**				**TCC (1) + DMA (2), X_1_ = 0.0564**			
293.15	0.963793	1535.12	1.427914	0.55827	0.962332	1569.88	1.435618	2.24954
298.15	0.959057	1515.99	1.425728	0.57120	0.957682	1549.70	1.433703	2.27238
303.15	0.953329	1496.72	1.423548	0.57039	0.952894	1527.68	1.431822	2.25815
308.15	0.949526	1477.44	1.421315	0.59063	0.948195	1507.23	1.429601	2.27499
313.15	0.943774	1458.23	1.419066	0.58812	0.943469	1486.86	1.427330	2.29182
318.15	0.939945	1439.05	1.416875	0.60845	0.938710	1466.52	1.425094	2.30651
323.15	0.935123	1419.86	1.414695	0.61512	0.933926	1446.30	1.422831	2.32331

X_i_ is molar fraction of TCC solute in binary mixtures. Standard uncertainties, u, are as follows: u(T) = 0.02 K and u(p) = 0.04 MPa, and the combined expanded uncertainty Uc in mole fraction, density, speed of sound and refractive index was Uc(x) = 0.0008, Uc(ρ) = 0.004 g cm^−3^, Uc(u) = 1.2 m s^−1^ and Uc(n_D_) = 0.0005, respectively (0.95 level of confidence).

**Table 3:** Calculated values of acoustic impedance, (Z); adiabatic compressibility, (κ_S_); space-filling factor, (S); specific refraction, (r_D_); relaxation strength, (r); intermolecular free length, (L_f_); surface tension, (σ); and modified surface tension, (σ_mod_), for pure solvents and binary TCC (1) + solvent (2) mixtures at various temperatures.

T/K	Z/10^5^ kg m^−2^s^−1^	κ_S_/ 10^−9^m^2^ N^−1^	S	*r_D_*/10^−3^ m^3^ kg^−1^	*r*	L_f_·10^10^/m	*σ*/N·m^−1^	*σ*_mod._/N·m^−1^
**TCC (1) + AE (2), X_1_ = 0**								
293.15	9.17468	0.93830	0.22149	0.28044	0.47290	0.62429	0.0197	0.0208
298.15	8.98144	0.97375	0.22039	0.28058	0.48929	0.64183	0.0191	0.0203
303.15	8.81246	1.00583	0.21918	0.28059	0.50282	0.65826	0.0186	0.0199
308.15	8.61345	1.04690	0.21811	0.28081	0.51960	0.67763	0.0181	0.0194
313.15	8.45382	1.08055	0.21719	0.28125	0.53187	0.69460	0.0176	0.0190
318.15	8.27316	1.12164	0.21588	0.28120	0.54636	0.71396	0.0171	0.0185
323.15	8.08371	1.16535	0.21477	0.28203	0.55983	0.73414	0.0166	0.0181
**TCC (1) + AE (2), X_1_ = 0.0626**								
293.15	9.50569	0.89032	0.22194	0.27588	0.45462	0.60812	0.0206	0.0217
298.15	9.31198	0.92276	0.22081	0.27596	0.47095	0.62480	0.0200	0.0212
303.15	9.14342	0.95193	0.21962	0.27596	0.48438	0.64038	0.0195	0.0208
308.15	8.94332	0.98945	0.21850	0.27610	0.50114	0.65878	0.0189	0.0203
313.15	8.78301	1.02002	0.21760	0.27655	0.51331	0.67487	0.0185	0.0199
318.15	8.60085	1.05746	0.21630	0.27651	0.52777	0.69324	0.0180	0.0195
323.15	8.42691	1.09494	0.21520	0.27677	0.54118	0.71162	0.0175	0.0190
**TCC (1) + nP (2), X_1_ = 0**								
293.15	9.84975	0.82904	0.23438	0.29140	0.41419	0.58682	0.0217	0.0229
298.15	9.65634	0.85840	0.23328	0.29145	0.43147	0.60261	0.0211	0.0224
303.15	9.48040	0.88592	0.23215	0.29156	0.44625	0.61778	0.0206	0.0220
308.15	9.30677	0.91496	0.23104	0.29154	0.46128	0.63349	0.0201	0.0215
313.15	9.13914	0.94420	0.22990	0.29152	0.47541	0.64930	0.0196	0.0211
318.15	8.95641	0.97828	0.22873	0.29146	0.49118	0.66678	0.0191	0.0207
323.15	8.78212	1.01274	0.22754	0.29131	0.50619	0.68439	0.0186	0.0202
**TCC (1) + nP (2), X_1_ = 0.0663**								
293.15	10.20069	0.78351	0.23447	0.28759	0.38849	0.57048	0.0227	0.0240
298.15	10.00963	0.80961	0.23335	0.28767	0.40520	0.58524	0.0222	0.0235
303.15	9.81870	0.83710	0.23228	0.28782	0.42177	0.60052	0.0216	0.0230
308.15	9.62887	0.86590	0.23114	0.28791	0.43808	0.61628	0.0210	0.0225
313.15	9.44016	0.89610	0.23001	0.28803	0.45413	0.63254	0.0204	0.0220
318.15	9.25290	0.92771	0.22885	0.28813	0.46987	0.64931	0.0199	0.0216
323.15	9.06647	0.96095	0.22757	0.28810	0.48538	0.66666	0.0194	0.0211
**TCC (1) + nB (2), X_1_ = 0**								
293.15	10.19180	0.77967	0.24183	0.29861	0.38136	0.56908	0.0228	0.0240
298.15	10.01511	0.80442	0.24078	0.29842	0.39816	0.58336	0.0222	0.0236
303.15	9.83551	0.82911	0.23964	0.29878	0.41259	0.59764	0.0217	0.0231
308.15	9.65496	0.85689	0.23853	0.29862	0.42930	0.61306	0.0211	0.0227
313.15	9.48272	0.88459	0.23743	0.29850	0.44484	0.62847	0.0206	0.0222
318.15	9.29909	0.91483	0.23632	0.29873	0.46024	0.64479	0.0201	0.0218
323.15	9.11079	0.94731	0.23519	0.29910	0.47560	0.66191	0.0195	0.0213
**TCC (1) + nB (2), X_1_ = 0.0697**								
293.15	10.35178	0.76121	0.24193	0.29658	0.37090	0.56230	0.0232	0.0245
298.15	10.16459	0.78574	0.24083	0.29666	0.38763	0.57655	0.0227	0.0240
303.15	9.97785	0.81149	0.23976	0.29677	0.40417	0.59126	0.0221	0.0236
308.15	9.79239	0.83838	0.23862	0.29682	0.42044	0.60641	0.0215	0.0231
313.15	9.60861	0.86643	0.23752	0.29693	0.43639	0.62198	0.0210	0.0226
318.15	9.42619	0.89573	0.23642	0.29705	0.45206	0.63802	0.0204	0.0221
323.15	9.24453	0.92648	0.23529	0.29716	0.46750	0.65459	0.0199	0.0217
**TCC (1) + THF (2), X_1_ = 0**								
293.15	11.56071	0.66409	0.24634	0.27755	0.33726	0.52521	0.0263	0.0277
298.15	11.28112	0.69315	0.24489	0.27762	0.36114	0.54151	0.0254	0.0270
303.15	10.99727	0.72477	0.24352	0.27782	0.38513	0.55878	0.0245	0.0262
308.15	10.71755	0.75839	0.24209	0.27791	0.40873	0.57675	0.0237	0.0254
313.15	10.44738	0.79311	0.24070	0.27806	0.43104	0.59508	0.0229	0.0246
318.15	10.16949	0.83174	0.23928	0.27818	0.45401	0.61481	0.0220	0.0239
323.15	9.89499	0.87293	0.23778	0.27821	0.47643	0.63539	0.0212	0.0231
**TCC (1) + THF (2), X_1_ = 0.0390**								
293.15	11.59803	0.66111	0.24653	0.27722	0.33558	0.52403	0.0264	0.0278
298.15	11.31807	0.68998	0.24504	0.27724	0.35947	0.54027	0.0255	0.0271
303.15	11.03721	0.72105	0.24361	0.27734	0.38325	0.55734	0.0246	0.0263
308.15	10.75941	0.75399	0.24224	0.27752	0.40646	0.57508	0.0238	0.0255
313.15	10.48520	0.78889	0.24083	0.27768	0.42909	0.59350	0.0230	0.0248
318.15	10.21550	0.82574	0.23944	0.27787	0.45102	0.61259	0.0222	0.0240
323.15	9.94838	0.86498	0.23806	0.27809	0.47247	0.63249	0.0214	0.0233
**TCC (1) + DMF (2), X_1_ = 0**								
293.15	14.08366	0.47885	0.25859	0.27226	0.14111	0.44598	0.0342	0.0360
298.15	13.82499	0.49443	0.25741	0.27238	0.16399	0.45735	0.0333	0.0353
303.15	13.57400	0.51023	0.25622	0.27254	0.18565	0.46883	0.0325	0.0346
308.15	13.31699	0.52741	0.25501	0.27264	0.20813	0.48097	0.0317	0.0339
313.15	13.07243	0.54448	0.25381	0.27278	0.22895	0.49306	0.0309	0.0333
318.15	12.82179	0.56317	0.25258	0.27281	0.25083	0.50591	0.0301	0.0326
323.15	12.57901	0.58210	0.25136	0.27290	0.27142	0.51886	0.0293	0.0319
**TCC (1) + DMF (2), X_1_ = 0.1261**								
293.15	14.79538	0.44028	0.25723	0.26689	0.07946	0.42764	0.0365	0.0385
298.15	14.53921	0.45369	0.25608	0.26701	0.10226	0.43810	0.0357	0.0378
303.15	14.26867	0.46825	0.25493	0.26741	0.12493	0.44913	0.0348	0.0371
308.15	14.02868	0.48247	0.25376	0.26725	0.14733	0.46002	0.0340	0.0364
313.15	13.76240	0.49829	0.25257	0.26762	0.16936	0.47169	0.0331	0.0357
318.15	13.52628	0.51374	0.25141	0.26747	0.19107	0.48319	0.0323	0.0350
323.15	13.27744	0.53044	0.25026	0.26762	0.21250	0.49530	0.0315	0.0343
**TCC (1) + DMA (2), X_1_ = 0**								
293.15	13.89953	0.48709	0.26256	0.27901	0.14782	0.44981	0.0337	0.0355
298.15	13.64346	0.50308	0.26133	0.27906	0.17085	0.46133	0.0328	0.0348
303.15	13.38944	0.51978	0.26005	0.27907	0.19352	0.47320	0.0320	0.0341
308.15	13.13724	0.53725	0.25880	0.27911	0.21586	0.48543	0.0312	0.0334
313.15	12.88758	0.55549	0.25759	0.27920	0.23779	0.49802	0.0303	0.0327
318.15	12.64101	0.57448	0.25641	0.27932	0.25929	0.51096	0.0296	0.0320
323.15	12.39618	0.59437	0.25521	0.27942	0.28044	0.52430	0.0288	0.0313
**TCC (1) + DMA (2), X_1_ = 0.0564**								
293.15	15.10746	0.42164	0.26127	0.27149	0.03730	0.41849	0.0377	0.0398
298.15	14.84120	0.43479	0.26026	0.27177	0.06189	0.42888	0.0368	0.0390
303.15	14.55717	0.44967	0.25928	0.27210	0.08836	0.44013	0.0358	0.0382
308.15	14.29148	0.46424	0.25812	0.27222	0.11260	0.45125	0.0350	0.0375
313.15	14.02806	0.47944	0.25692	0.27232	0.13642	0.46268	0.0341	0.0367
318.15	13.76637	0.49533	0.25575	0.27245	0.15989	0.47446	0.0332	0.0360
323.15	13.50737	0.51188	0.25456	0.27257	0.18290	0.48656	0.0324	0.0353

X_i_ is molar fraction of TCC solute in binary mixtures.

**Table 4:** The physicochemical parameters of TCC in organic solvent solubility (s) and constant (K_OW_) values at 298.15 K and at pressure p = 0.1 MPa.

Solvent Name	X_1_	Solubility TCC in Solvent	K_OW_
Ethyl alcohol	0.062619	1.138753	0.173696
n-Propyl alcohol	0.066311	0.945736	0.184112
n-Butyl alcohol	0.069708	0.815611	0.192859
Tetrahydrofuran	0.038987	0.496207	0.225380
N,N-Dimethylformamide	0.126069	1.864863	0.148805
N,N-Dimethylacetamide	0.056367	0.642128	0.207878

**Table 5:** Specification of triclocarban, Ethyl alcohol, n-Propyl alcohol, n-Butyl alcohol, Tetrahydrofuran, N,N-Dimethylformamide and N,N-Dimethylacetamide compounds used in samples.

Chemical Name	Molar Mass/g mol^−1^	Supplier	Mass Fraction Purity/%	Purification Method *
3,4,4′-Trichlorodiphenylurea	315.58	Sigma Aldrich	≤100%	dried in vacuum
Ethyl alcohol	46.070	Sigma Aldrich	≥98%	none
n-Propyl alcohol	60.100	Sigma Aldrich	≥98%	(G.C.)
n-Butyl alcohol	74.120	Sigma Aldrich	≥99%	none
Tetrahydrofuran	72.110	Fluka Chemie AG	≥99%	none
N,N-Dimetilformamide	73.090	E. Merck	99%	none
N,N-Dimethylacetamide	87.120	Fluka Chemie AG	>99.5%	none

* The purity of these compounds was analyzed by the suppliers.

## Data Availability

Data are contained within the article.
